# Age-related differences of tooth enamel morphochemistry in health and dental caries

**DOI:** 10.1186/s13167-014-0025-8

**Published:** 2015-01-29

**Authors:** Anatoly A Kunin, Anna Yu Evdokimova, Natalia S Moiseeva

**Affiliations:** Department of Therapeutic Dentistry, Faculty of Dentistry, Voronezh N.N. Burdenko State Medical Academy, Avenue of Revolution Str. 14, Voronezh, Russia

**Keywords:** Dental caries, Tooth structure, Age-specific differences of teeth, Morphochemical features, Enamel

## Abstract

Currently, dental caries is the main reason of patient visits to dentists. A great deal of scientific work is dedicated to the study of enamel caries. The reason for this is the necessity for more detailed study of the pathogenesis of dental caries and other pathological processes occurring in tooth enamel. The application of modern high-technological methods of research has made it possible to study enamel structure in detail. Hard dental tissues are composed of organic and inorganic components and water. The organic substance consists of proteins, lipids, and carbohydrates. At different ages, caries intensity may vary. The carious process often develops during the first months after the tooth eruption but much less in adulthood and old age. These processes are mainly associated with the mechanisms of ionic exchange between the oral cavity and hard dental tissues. Different groups of teeth are differently affected by the carious process. Previous studies have revealed that age is an important factor influencing on the structure and chemical composition of hard dental tissues. Various recent works at the Department of Therapeutic Dentistry of Voronezh N.N. Burdenko State Medical Academy (VSMA) have studied not only the structural-morphological features of the enamel in children and adults but also the level of metabolic processes inside it. As a result of aging, teeth change both the enamel structure and the level of its ionic processes, but unfortunately, these changes have not been well characterized in teeth with conventional age-specific differences.

## Review

### Introduction

Currently, dental caries and its complications are the main reason of patient visits to dentists [1-4]. This fact highlights the relevance of the problem, both in the medical and social spheres.

The results of the national epidemiological dental survey conducted by the WHO criteria in 2007–2008 [5] indicated that the prevalence of dental caries remained high among the population of the Russian Federation: caries of primary teeth was found in 84% of children aged 6, caries of permanent teeth was in 72% of children aged 12 and in 99%–100% of adults. It is interesting to compare the main incidence for dental diseases among the population in the years 1998 and 2008. Special attention is given to children due to the fact that caries development is present both in primary and permanent dentitions. The analysis of the incidence rates in childhood demonstrated the stability of the average intensity of primary tooth caries in 6-year-old children over the 10-year period. There was a redistribution of the components in the decayed, missing, filled teeth (DMFT) caries index structure, namely: a 15% reduction in the number of teeth affected by caries and 22% increases in the number of filled teeth. The number of extracted primary teeth became 2.5 times higher in 1998. At the same time, the prevalence of dental caries in permanent teeth in 6-year-old children reduced by 9%, and in the age groups of 12 and 15, by 6%. The average DMFT caries index in children aged 6 over the 10-year period did not change, while in children aged 12 and 15, it fell by 13.7% and 12.8%, respectively. The level of caries intensity in 12-year-old children in 2008 was assessed as low, in accordance with the WHO grading scale. In both groups, the value of the D component (decayed teeth) decreased by 28%, the number of filled teeth increased, and the number of extracted teeth became 2–3 times less compared to 1998. Over the same 10-year observation period, the number of extracted teeth in patients aged 35–44 decreased by 14%. In the age group 65 or older, the average caries intensity over the 10-year period did not change significantly: the E component (extracted teeth) [1] made up about 80% of the DMFT caries index structure both in 1998 and 2008.

The low level of personal hygiene and lack of motivation for dental diseases prevention and good oral hygiene habits among the population are typical for the greater part of the population [2,6].

In our opinion, poor oral hygiene can occur at any age, both in young and old people, and can be defined after a dental examination. In the contrast, systemic diseases affecting the balance between the demineralization and remineralization processes, finally resulting in dental caries formation, are much less common for the younger group. Thus, the age-related particularities of dental caries formation should be taken into consideration when developing dental caries predictive and preventive measures.

The national epidemiological dental survey showed a 6%–13% reduction of dental caries intensity in children over the 10-year period [1]. It seems to us that such a favorable situation became possible due to increased attention to oral hygiene through the promotion of healthy lifestyle among children. It should be noted that the reduction in caries was much less pronounced in elderly patients.

#### Structural features of tooth enamel

In international and Russian dental literature, a special emphasis is placed on the study of the microstructure and physiology of hard tooth tissues. A great deal of scientific work is dedicated to the study of enamel [6,7]. The reason for this, primarily, is the necessity for more detailed study of the pathogenesis of dental caries and other pathological processes occurring in tooth enamel.

Enamel is the only tissue of ectodermal origin with an acellular structure, exposed to calcification in the absence of blood vessels and nerves. According to current concepts, tooth enamel is the hardest tissue in the human body due to the high content of inorganic substances (97%) [3]. Enamel proportions are rather small: its thickness at the neck—0.1 mm, and on the occlusal surface—1.62–1.70 mm. According to the data of other research, the thickness of the enamel layer is maximal in the area of chewing tubercles of permanent teeth, where it reaches a thickness of 2.3–3.5 mm; on the lateral surfaces of permanent teeth it is usually 1–1.3 mm thick, and it does not exceed 1 mm in primary teeth. The thinnest layer of enamel (0.01 mm) covers the neck of the tooth [3]. According to the data obtained at the Department of Therapeutic Dentistry of Voronezh N.N. Burdenko State Medical Academy, the enamel thickness on chewing tubercles of buccal teeth ranges from 1.5 to 1.8 mm, equals 0.2 mm at the neck of posterior teeth, and decreases to zero at the cement-enamel junction [8].

Thus, various sources give different values for enamel thickness, indicating variations. An important aspect to consider is the tooth age, but it has not been touched upon in the available literature.

The structural features of enamel became known in 1841, when it was firstly described that its structure consists of hydroxyapatite crystals. The smallest structural units of enamel are apatite crystals. They differ from other hard tissue crystals by its larger size (1,600 × 400 angstroms (A) compared to 640 × 40 A for bone tissue) [3]. Each hydroxyapatite crystal is covered by a hydration shell about 1 nm thick. Most enamel crystals are rod-shaped, but there are also needle-like, cube-shaped, rectangular, rhombohedral and other crystals. The crystals combine to form a larger structure—enamel prisms. Each prism has conventional component parts such as an arcade-shaped head and an appendix, which is wedged between two subjacent prisms [9].

According to a number of literature sources, there are various forms of prisms and a variety of options regarding their orientation to each other [10].

A prism head can appear above the enamel surface, lie on the same level slightly contouring, or be a cavity. In the last case, it gives to enamel a honeycomb appearance. The prism diameter averages between 4.0 and 7.5 μm [3]. The prisms, in their turn, are also characterized by different orientation of crystals. In the central section of the prism, they are arranged in straight lines and coincide with the longitudinal axis, but gradually, the crystal orientation is changing, and in the peripheral areas, they are located at a small angle to the prism surface [11].

It had been previously believed that each prism had a cover containing a large amount of organic matter. Later, with the use of more advanced techniques, including electron microscopy, it was found that the enamel interprismatic substance consists of the same crystals as the prism itself but differs by their orientation [12].

The most recent data indicate that the enamel tubules and interprismatic enamel spaces are filled with protein biopolymers and hyaluronic acid [13].

The work by A.A. Kunin using electron microscopy suggests that between the enamel prism tufts, there are S-shaped curving tunnels up to 3 μm in diameter, which open on the enamel surface [14].

**Figure 1:**
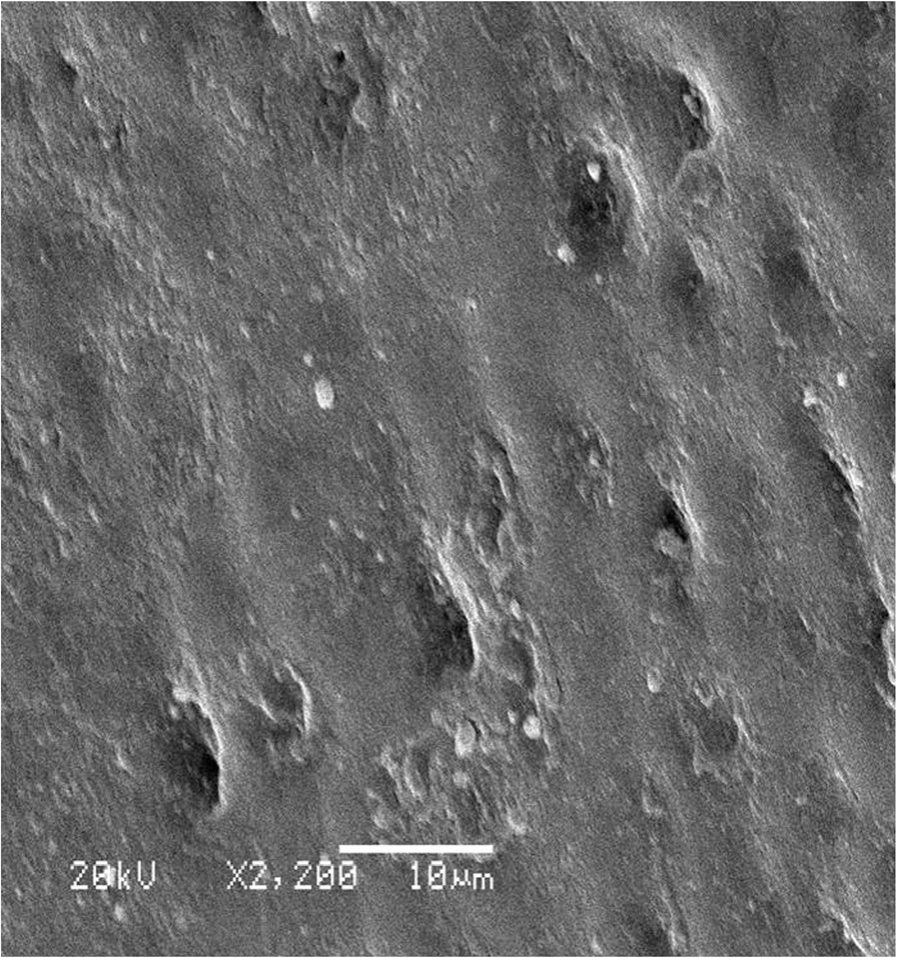
**Enamel tubules and cavities on the surface enamel layer.**

**Figure 2:**
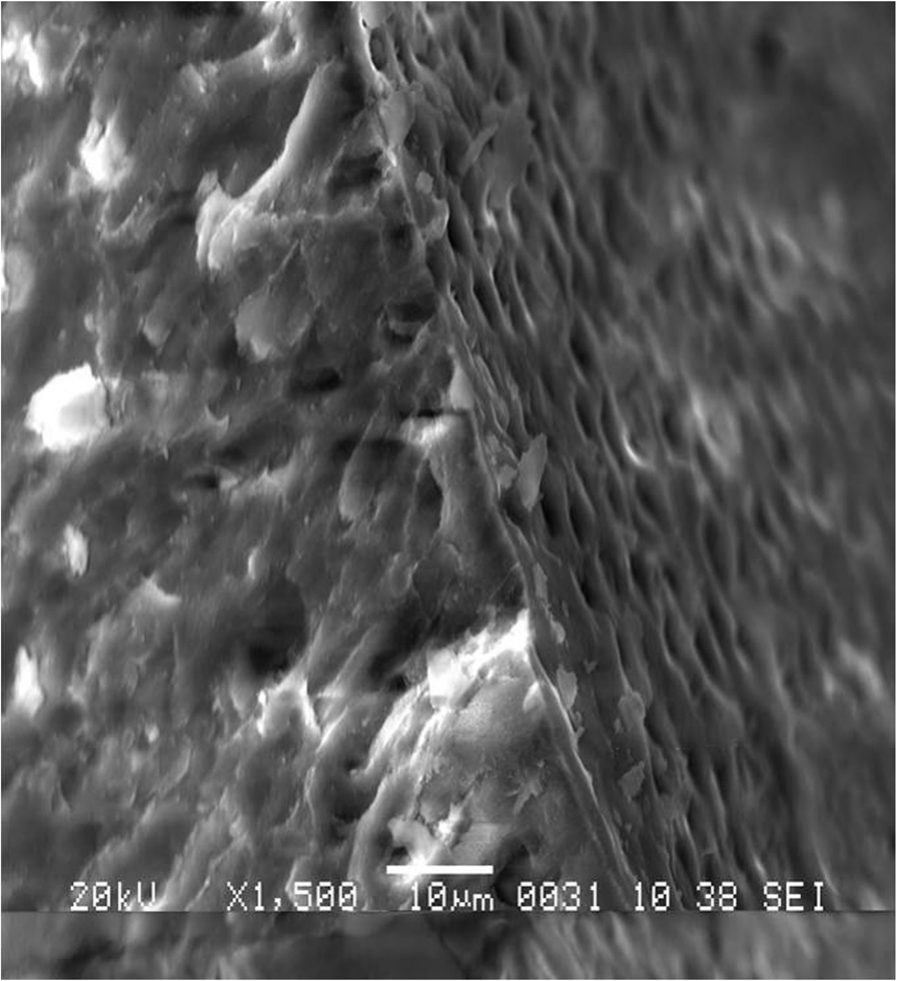
**Openings of enamel tubules from surface layer through the enamel prisms.**

In the enamel, there are areas with poorly calcified interprismatic substance: enamel lamellae, tufts, and spindles (Figures 1 and 2). They consist mainly of organic compounds in a form of thin fibrous structures [9,11,15].

The enamel lamellae resemble thin leaf-like structures that extend from the enamel surface to the enamel-dentine junction. They extend in a longitudinal direction and are more easily detected in the transversal (longitudinal) plane of the tooth where they look like fine cracks. As it has been already mentioned, the research work of the Department of Therapeutic Dentistry of Voronezh N.N. Burdenko State Medical Academy (VSMA) proved that they are not cracks but enamel tunnels. They serve for easier penetration of organic substances into the enamel [14,16,17].

Before the enamel tunnels were identified, many authors had held the view that the enamel lamellae were cracks in the enamel filled with enamel proteins and organic debris. As enamel lamellae may be detected even in pre-eruptive teeth, their presence is considered to be due to deviations in the process of enamel development. After the tooth erupts, a number of enamel lamellae increase as a result of various physical and chemical influence on the enamel [17,18].

As mentioned above, it has been proven that the amount of enamel tunnels may only decrease with age as a result of enamel attrition and closing of enamel openings in case the attrition reaches a tunnel bending. In this case, such an enamel tunnel may look like cracks [19].

Thus, the enamel tunnels are located between the enamel tufts, which extend from the enamel-dentine junction and penetrate into about one-third of the enamel thickness, although may reach the enamel surface in the cervical area. It has been found that the enamel tufts are long ribbons of an organic substance, which grow out of the enamel-dentine junction and are oriented along the tooth crown. The enamel tufts are similar to the enamel lamellae to which they are in parallel. The base of such a ribbon-like branch is a straight line along the enamel-dentine junction, and its free end in the enamel coincides with the prisms direction [18,20].

In the longitudinal plane of the tooth, some flask-shaped, structures, thickened at one end, called enamel spindles, were seen. They extend from the enamel-dentine junction and penetrate into about one-third of the enamel thickness [21].

The spindles look like thin “blind-ended” canals, which contain side branches of the odontoblastic processes or their degradation products. The tooth slices where the spindles and the dentinal tubules are arranged in one plane show that they are continuations of each other. Their formation is associated with the interpenetration of odontoblastic processes into the layer of enameloblasts or, vice versa, enameloblastic processes into the dentin matrix [22]. Such a detailed description of enamel structure provides new evidence concerning the tubular structure of enamel including the enamel-dentine junction that has been obtained in recent years [4].

We consider the presence of enamel bridges in dentin, through which the odontoblastic processes penetrate into enamel, the most probable variant of the mentioned enamel areas structure. This is confirmed by numerous recent works [4,16,23,24].

The enamel surface may be represented by prismatic and non-prismatic areas. In the prismatic areas, we see well-defined enamel prism forms: arch-shaped, arcade-shaped, or irregularly shaped one. In the non-prismatic enamel areas, single ridge-shaped prisms may be found. The reviewed research has not revealed any regularity in the non-prismatic arrangement [16,25].

There are some data describing several types of the tooth enamel structure [15].

Type 1 is defined as having a homogeneous coating, which is an organic cover of the enamel. The organic substance has a dense structure with the enamel prisms not clearly outlined; this structure corresponds to the areas of non-crystalline enamel.

Type 2 is characterized by zones where the organic coating only fragmentarily covers a subjacent inorganic component resulting in tissue scalloping. The enamel prism contours may be observed on the surface.

Type 3 is characterized by absence of the organic coating on the enamel surface and clearly defined enamel prism contours.

Type 4 is characterized by a homogeneous organic coating on the enamel surface with slightly pronounced enamel prism contours, which give a wave shape to the pattern.

Different types of the enamel structure determine unequal resistance of the enamel surface layer to various external factors including dental caries progression [11].

It is necessary to pay attention to some unique features of the enamel structure in different parts of the tooth [26]. In the contact points, the enamel surface is flat; it includes prismatic and non-prismatic sites. In the area between the tooth neck and its survey line, there are Retzius lines exit points (perikymats). Above the survey line, towards the cusps and cutting edges, they are less visible. The perikymats appear as uneven curving lines encircling the tooth surface [16,27].

Borovskij referred to Hoffmann [28] who distinguished two types of perikymats: undulating and overlapping. Type 1 is more common for erupted teeth, and type 2 is more typical for non-erupted teeth. Based on these data, the author suggested that different types of perikymats reflect degrees of enamel maturity.

The electron microscopy of non-erupted teeth shows that most parts of the enamel surface have a prismatic structure, which becomes even more evident after acid-etching of the enamel [3]. Gradually, in the process of post-erupting maturation, the enamel surface acquires a non-prismatic structure due to the increased surface mineralization. Thus, prismatic areas of the enamel surface are more frequent in teeth of younger patients. In non-prismatic areas, the apatite crystals are placed at right angle to the enamel surface, which results in its fine-grained appearance [29,30].

The formation of these areas comes from a compensatory response to the enamel attrition. The first mention of the fact that there are cavities in the enamel was firstly recorded in 1841. The staff of the Department of Therapeutic Dentistry of VSMA developed a method of combined scanning electron microscopic study of hard dental tissues by increasing modification up to 50,000 times. The results achieved with the use of this technique showed a number of morphological features of enamel [8,14,31,32]. Rounded formations on the enamel surface 3 μm in diameter are beginnings of “tunnels” with a diameter ranging from 1 to 3 μm, which extend through the entire thickness of the enamel and have an S-shaped curvature along the enamel prisms. It should be noted that the number of rounded formations in primary teeth 1–2 μm in diameter is high, and they are characterized by being honeycomb-shaped and uniformly distributed over the enamel surface. Their number decreases with age; their distribution on the enamel surface becomes less; and the diameter increases up to 3 μm. In some parts of the enamel-dentine border, the enamel penetration into dentin reaches a depth of 5–8 μm and a width of 5–8 μm with the “enamel tunnels” components and structures conserved. These formations are called “enamel bridges”. Each “enamel bridge” has 5–7 “enamel tunnels”. Their density is higher than in the enamel mass but their diameter is a bit smaller and equals 0.15–0.5 μm. These structural formations are involved in maturation processes of the enamel and dentine, oral fluid, and filling materials [33,34].

Recent results of tunneling microscopy of enamel and dentin do not only confirm the presence of the previously identified features of the tooth microstructure but also reveal the presence of “tunnels” of the first, second, third, and fourth orders. They define more precisely the qualitative and quantitative microelement structure of their walls and inner content of both organic and inorganic nature [14,35].

On the basis of the X-ray microchemical analysis and histochemical studies, it has been found that the enamel tubules and interprismatic enamel spaces are filled with protein biopolymers and hyaluronic acid [16].

Relying upon the scientific data obtained over the last few years, it can be concluded that the understanding of enamel structure and its inner processes is changing in some respects, which encourages its further detailed study.

#### Chemical composition of normal enamel

The use of modern high-technological methods of research has made it possible to study the enamel structure in details. Hard dental tissues are composed of organic and inorganic components and water. The organic substance consists of proteins, lipids, and carbohydrates. These are the following fractions in enamel proteins: acid-soluble–0.17%, acid-insoluble–0.18%, peptides and free amino acids–0.15%, lipids–0.6%, and citrates–0.1% [36].

Lately, special attention has been paid to the organic components of the tooth enamel. According to some studies, the protein content in the enamel ranges between 0.5% and 3%–4%, which is only a small fraction of the entire mass. That is the reason why the role of protein in the physiological processes of enamel including its calcification has not been duly considered. The improvement of methods for obtaining pure enamel offers the opportunity of its detailed study. In this connection, the opinion has arisen that caries resistance of enamel depends on the content of both inorganic substances and proteins [37]. A number of studies have revealed a significant difference in the amino acid composition of collagen proteins and enamel. Enamel proteins contain significantly fewer amino acids, which are more specific for collagen.

The fact that there is a liquid in hard dental tissues was first mentioned in 1928. The research, which followed, revealed that the fluid in the enamel exists in two forms: free water located in micro spaces and hydration shells of apatite crystals. The total volume of water in the enamel is 3.8% [12].

The inorganic part of enamel consists of hydroxyapatite (75%), carbonate apatite (19%), chlorapatite (4.4%), fluorapatite (0.66%), and non-apatite forms (2%) [3]. The basic structural units of a prism are considered to be crystals of apatite origin with hydroxyapatite as the main one. Each hydroxyapatite crystal is coated with a hydration shell [38]. Its thickness is approximately 1 nm and the crystals are located 2.5 nm apart [15]. The apatites determine the enamel strength. The composition of apatites may range significantly. The ideal hydroxyapatite composition corresponds to the formula Ca_10_ (PO_4_)_6_ (OH)_2_, where the Ca/P molar ratio is 1.67. However, in case Ca is substituted by other microelements, the Ca/P ratio may vary within the limits from 1.33 to 2.0. Thus, in case Ca is substituted for similar chemical elements such as Ba, Cr, Mg, the Ca/P index ratio is reduced to 1.33 and the properties of the obtained apatite are changed in regard of the enamel resistance to an adverse environment [39].

The hydroxyl groups’ substitution by fluoride results in fluorapatite formation, which exceeds the “ideal” hydroxyapatite in its strength, and the Ca/P ratio can be increased to 2.0. The Ca/P index is always used as a criterion of caries resistance of hard dental tissues, as hydroxyapatite crystals are characterized by the ability of physical-chemical exchange [25].

The latest technical developments seem to provide more reliable data for scientific research. According to the X-ray microanalysis results of the recent reports, the content of microelements in the intact enamel has been determined (Table 1) [34,40,41].

**Table 1:** **Content of microelements in the intact enamel (X-ray microanalysis, weight %, mean ± SD)**

**Microelements**	**Content of elements**
Na	0.79 ± 0.05
Mg	0.22 ± 0.01
K	0.21 ± 0.07
Ca	26.41 ± 2.25
Si	0.17 ± 0.04
P	16.01 ± 0.89
S	0.14 ± 0.07
F	0.14 ± 0.004
Cl	0.15 ± 0.02

The most important role is obtained by Ca and P as the main elements constituting the enamel. In the normal exchange process, the Ca/P ratio is 1.67, but later studies have shown that the coefficient increase up to 1.8 is considered to be more favorable [24].

#### Enamel content of microelements

While studying the chemical composition, many scientists put emphasis on the significant differences between the outer and inner layers of the enamel. On the basis of the data received by Russian scientists, the highest Ca and P contents are registered in the surface layer of the intact enamel; in the middle layers, the content of the elements is a bit lower, and it is the lowest at the enamel-dentine border [28,36].

Apart from these elements (Table 2), the enamel contains a number of other microelements [29].

**Table 2:** **Content of Ca and P in various layers of the enamel**

	**Ca**	**P**
Surface layer	39.83 + −0.14%	18.53 + 0.08%
Middle layer	39.53 + −0.13%	18.16 + −0.07%
Enamel-dentine border	39.07 + −12%	17.92 + −0.07%

Enamel mineralization is mainly determined by its Ca and P contents that are non-uniform in different teeth and particular tooth areas. The studies conducted on the second upper molars have revealed that the contents of MgO, Na_2_O, K_2_O, and F significantly increase from the surface to deeper layers of the enamel, while the contents (wt. %) of P_2_O_5_ and CaO show a decrease [6,7].

Enamel also contains such elements as Mn, Fe, Ni, Co, Zn, Cu, Pb, Sr, and Hg. The concentration of Mg and Sr increases towards the enamel-dentine border, while the contents of Zn, Cu, Pb, Mn, and Fe fall as the enamel-dentin border was approached. The Pb content increases with age; it is considered to substitute for calcium in the enamel hydroxyapatite [42].

T.A. Smirnova [43] reported the content of microelements in the enamel of teeth of different groups. The author indicates a decrease in the Ca and P contents in the cervical area of the vestibular surface of premolars and molars and no similar changes in incisors and canines.

The Ca, P, and F contents in the surface layer of enamel increase after the tooth eruption and during the first year because of saliva maturation from the enamel [44].

In many research reports [23], the Ca/P ratio had been assessed mainly through the method of acid biopsy of enamel, which was followed by more advanced techniques.

To determine the content of various elements in the samples, the method of atomic emission spectroscopy was used. The intact enamel composition of 28 elements was determined. The study revealed the following data: in all the samples, regardless of their tooth decay resistance level, the distribution of elements was the same but their content was different. For example, the relative intensities of calcium, phosphorus, cuprum, saturn, magnesium, manganese, sodium, cobalt, and sulfur in the enamel samples with a high caries resistance level were higher in comparison with the enamel samples with a lower caries resistance. The relative intensities of iron, zinc, boron, potassium, silicon, and strontium in the enamel samples with a high resistance level were, in contrast, lower [45].

The data obtained at the Department of Therapeutic Dentistry of VSMA have revealed the following results: the highest Ca and P content is observed in the area most vulnerable to dental caries, the area of fissures, and in the cervical area, a place of the greatest accumulation of plaque; the most caries resistant place is the incisor edge [34,46].

With the use of X-ray microchemical analysis, the composition of chemical elements on the enamel surface and at the openings of the intact enamel tubules has been identified [13].

The enamel surface layer turns out to be more saturated with mineral elements, in contrast with a deeper layer of the enamel. The distribution of extracted teeth by age group reveals reliable differences in the content of chemical elements. At the opening of the enamel tubule, the C content is 19.07 ± 0.23; the N content is 6.28 ± 0.09, and the O content is 32.86 ± 0.34 in the 16–18 age group. By 55–60 years, the concentration of these elements falls to 11.87 ± 0.17, 3.42 ± ± 0.08, and 24.96 ± 0.53, respectively. At the same time, the Cl content increases from 0.6 ± 0.011 to 0.79 ± 0.017; the Ca content—from 32.03 ± 0.51 to 36.7 ± 0.06; the P content—from 15.4 ± 0.37 to 18.02 ± 0.33; the Mg content—from 0.21 ± 0.009 to 0.3 ± 0.007; and the Na content—from 0.59 ± 0.04 to 0.71 ± 0.02 [13,25,47].

#### Specific characteristics of the tooth enamel structure of humans and animals

The chemical composition analysis of enamel is extremely important in caries activity prediction. Therefore, it is interesting to study the enamel of animals, in which dental caries is rarely found.

In the course of study of specific characteristics of the tooth enamel structure of humans and caries-resistant animals, a number of significant differences have been revealed. Pigs and cows’ tooth enamel is characterized by fewer protein components, but their greater ability to accumulate calcium. This, in turn, provides higher mineralization of tooth enamel in animals than in humans [48].

After the comparative evaluation of human and beaver tooth enamel, the following data were obtained. The chemical composition of beaver enamel has an increased content of magnesium, sodium, potassium, and silicium and a decreased content of sulfur and chlorine. The beaver enamel microhardness is 1.53 times higher than that of humans. These figures indicate that beaver tooth enamel is functionally stronger [49]. That is how the extremely rare carious teeth in these animals can be explained. It also proves that fluorine is only needed in childhood as in the newborn beavers’ teeth (it is known that they are born with teeth), the F content is 10 times higher than in humans, and in adult beavers’ teeth, despite the absence of caries, only traces of fluoride are found [41].

These results should be considered not only for the development of methods for prevention and treatment of dental caries in humans but also for the assessment of changes in the ionic processes at different stages of life.

#### Morphochemical features of the enamel in dental caries

According to the World Health Organization, dental caries can be considered as the most widespread disease. The importance of the problem is confirmed by its involvement of all age groups [34]. This pathology often leads to a loss of teeth both in young and elderly people, which, in turn, results in a temporary disability of the working-age population [2,3,17].

At different ages, the caries intensity may vary. The carious process often develops during the first months after tooth eruption but is much less in adulthood and old age [44]. These processes are mainly associated with the mechanisms of ionic exchange between the oral cavity and hard dental tissues.

The isomorphic and isoionic exchange in enamel crystals occurs from the diffusion of substances through the interprismatic spaces into the hydroxyapatite crystals. Thus, the inorganic components, Ca^2+^, Mg^2+^, and other ions penetrate into the enamel. The ions are transmitted from the oral fluid. The organic components are likely to come through the structural formations and defects of enamel—micro cracks, enamel tufts, and lamellae. Another source of ion movement into the enamel is from the cementum through the cement-dentine border to the enamel-dentine border [22].

According to some authors, the rate of ion exchange depends on such factors as the volume of micro spaces and amount of water associated with the crystals, which is involved in the ionic exchange. The intercrystalline spaces create the possibility of exchange of bound water presented in the organic matter. Small molecules, in their turn, can penetrate through the intercrystalline spaces. The ionic exchange rate varies with age. Young, immature enamel is notable for its active exchange processes. The ion diffusion is provided by enamel and the presence of a significant hydration layer. A mature enamel has small intercrystalline spaces and, in this connection, is poorly adapted for ion exchange [3,10].

Different groups of teeth are differently affected by the carious process. The first and second molars, especially their fissures, are more often affected, but over time, the caries progresses on approximal surfaces of upper premolars, a lower second molar, upper canines, and incisors [44].

These data should be considered in the development of preventive measures. Initial caries detection causes certain difficulties in clinical therapeutic dentistry, especially when there is a “white spot” of small size, because the surface layer of enamel remains clinically intact. Chemical and thermal irritants do not cause subjective sensations of pain [38]. Therefore, over a number of years, special attention has been given to studying of the morphological features of early tooth decay. The research supported by different techniques has made it possible to reveal changes in the structure and chemical composition of enamel in “white spot” areas [17,47].

Depending on the degree of mineralization, caries without the cavity formation is divided into zones as follows: a translucent zone (spot area), a dark zone that separates the affected body from the translucent zone, and an intact surface layer [28]. It has been determined that the presence of these zones depends on micro spaces, which are formed in the enamel in the process of its demineralization caused by caries activity. D. Hastings referred to A. Darling [50] indicates that if normal enamel micro spaces constitute 0.5% of the enamel volume, they reach a volume of 25% in the enamel caries, 4%—in the dark zone, and 2%—in the translucent zone, which creates optimal conditions for substances penetration into the enamel.

When the spot reaches 3 mm^2^ in size, the organic matrix undergoes changes. This is followed by an increase in the organic content due to its increased uptake from oral fluids [27].

It is found that when a white spot area increases, its depth also increases. If the surface diameter of white spots is up to 3 mm, the changes affect ½ of the enamel thickness; in case it exceeds 5 mm at diameter, the spot reaches the enamel-dentine border; sometimes causing variations in dentine. During these processes, the surface layer remains unchanged [38].

Later studies by X-ray structural analysis showed that the most mineralized area is the surface layer of enamel that is 3 μm thick with a crystal lattice of apatites denser than in underlying layers [24].

The processes occurring at the early stages of tooth decay have been studied in detail. Two stages have been identified. The first stage is characterized by the destruction and fragmentation of enamel prism with the concurrent weakening of the intercrystalline bonds in enamel. The hydroxyapatite crystals are positioned randomly. The caries process develops mainly along the enamel prism borders. The second stage is characterized by an increase in the enamel crystal size and a transformation of their hexagonal configuration into the polygonal one [9].

The natural state of tooth enamel in life is a dynamic balance between the demineralization and remineralization processes, which violation may result in caries progression. Therefore, such factors as the degree of enamel remineralization are important criteria for dental caries resistance assessment. The process of enamel mineralization after the tooth eruption is called “maturation”. The maturation takes place throughout a person’s life. A great importance in the study of the enamel maturation is given to its permeability and solubility. There is no doubt that these parameters are changing during life [15].

It should be noted that the enamel permeability decreases with age due to the deposition of mineral substances in the enamel coming from the saliva. Reasoning from this fact, we can estimate the degree of enamel maturity in accordance with its permeability [44].

Many authors mention an increase in the enamel permeability in caries. It depends on several factors such as a composition of the oral fluid, pH of the saliva, and characteristic properties of a penetrating agent [28,49].

Dental plaque plays a significant role in changing enamel permeability. It contributes the penetration of mineral components into the enamel by increasing its permeability [27].

The data obtained in the study of microelement composition of intact enamel and enamel covered with dental plaque is shown in the following table (Table 3).

**Table 3:** **Microelement composition of intact enamel and enamel covered with dental plaque**

**Microelement composition**	**Intact enamel**	**Enamel covered with dental plaque**
Ca	37.45 ± 1.4%	26.01 ± 2.78%
F	0.98 ± 0.02%	0.72 ± 0.12%
P	17.08 ± 1.23%	12.18 ± 1.48%
Mg	0.74 ± 0.01%	1.7 ± 0.70%
Inorganic substances	95.46 ± 1.2%	51.23 ± 1.56%
Organic substances	3.56 ± 1.02%	48.71 ± 1.24%

The comparative evaluation of microelements in the intact enamel and the enamel covered with plaque has shown that plaque has a pronounced ability to demineralize the enamel, which results in a reduction of the inorganic substances by 44.23% and an increase of the organic matter by 45.15% compared to intact enamel. The reduction of mineral components was primarily due to calcium 11.45% and phosphorus 5.08%. According to the findings, the enamel solubility is not uniform even willing. The most soluble enamel is at the gingival area and proximal contact surface of the tooth, which indicates their reduced mineralization. The least soluble and most mineralized zone is the occlusal part of the enamel [39,51].

The level of enamel permeability is different at different ages. It is the highest in non-erupted teeth, and then it reduces in permanent teeth immediately after their eruption; primary teeth are less permeable, and permanent teeth of adults have the lowest permeability [44].

The changes in enamel permeability also depend on a type of teeth: incisors have the lowest permeability, and it gradually increases towards molars. As for the tooth surfaces, the oral surface is more permeable than the vestibular one [28].

A number of researchers have reported that, at the white spot stage, the enamel demineralization is more pronounced in the subsurface than in external layers. This is explained by the chemical composition of the surface layer, namely a high Ca and P content [30].

X-ray microanalysis of enamel caries has shown that the highest Ca and P concentration is observed in the surface layer—39.83 ± 0.14% and 18.53 ± 0.08%, respectively, and in the middle zone—39.53 ± 0.08% and 18.16 ± 0.07% by weight [9,52].

Recent studies conducted at the Therapeutic Dentistry Department of Voronezh State Medical Academy made it possible to identify the groups of chemical elements contained in intact and carious teeth, which determine pathological changes in the lesion. The intact enamel surface at the enamel tubules openings and the area affected by primary caries has been scanned with the use of X-ray microanalysis (Table 4) [16].

**Table 4:** **Content of microelements in the intact and carious enamel of teeth (weight %, mean ± SD)**

**Elements**	**Intact teeth enamel tubule (** ***n*** **= 30)**	**Carious teeth enamel tubule (** ***n*** **= 30)**
С	19.34 + −0.27	31.43 + −0.25
S	0.18 + −0.003	0.73 + −0.007
Cl	0.67 + −0.015	0.99 + −0.018
N	5.17 + −0.05	10.27 + −0.05
О	34.34 + −0.54	15.48 + −0.19
Na	0.62 + −0.03	0.5 + −0.01
P	19.02 + −0.41	14.21 + −0.25
Ca	31.97 + −0.60	25.04 + −0.31
Zn	0.17 + −0.009	0.15 + −0.001
К	-	0.41 + −0.05

These studies have shown an increase in the weight percent of C, S, Cl, and N and a reduction in the weight percent of O, Na, P, Ca, and Z in carious tooth enamel. The appearance of potassium at the enamel tubules openings and its absence in the intact enamel have been identified. The research has also resulted in detection of groups of factors. The first factor is composed of such elements as C, P, Ca, Cl, and S. The second factor is oxygen; the third one is sodium [13,16,53].

The major source in the pathogenesis of dental caries is a violation of the dynamic balance between the remineralization and demineralization processes [7]. In initial caries, the intensity of mineralization is reduced, which is evident in the reduction of microelements. The composition of the surface enamel layer has been examined using the method of electron microprobe analysis. The obtained data are as follows: in the intact enamel, the Ca content is 37.39 + 2.61, the P content is 17.97+ 2.07, and the F content is 0.14 + 0.01 (in wt. %, M + m); in the white spot area, the Ca content is 13.79 + 1.86, the P content is 8.42+ 1.50, and the F content is 0.10+ 0.01 (in wt. %, M + m). In the white spot area, the Ca content is decreased almost threefold, the P content—twice, the F content—1.4-fold, which indicates a decrease in the mineralization intensity. The study of the demineralization intensity according to a rate of Ca and P output from the surface layer into the bioptate has shown that the initial caries is characterized by the reduction in the enamel dissolution rates by 25% for Ca and by 50% for P. The obtained data show that the demineralization processes, even less intensified, are prevalent over the remineralization processes, which is clinically proved by the demineralized carious spots. Consequently, the most important part of the pathogenesis is a reduction of the level of both phases of physical and chemical exchange in the enamel—remineralization and demineralization with the prevalence of the intensity of the latter [5].

#### Enamel dissolution

In the study of tooth decay etiology, the problem of enamel solubility is of great importance. Enamel dissolution is a uniform, stoichiometric process; its speed is determined by diffusion of the acid molecules from plaque fluid into the enamel surface. During the study of the solubility of the surface layer of primary and permanent tooth enamel in people of different ages, significant differences have been found. This indicates that this property of the enamel is constant and does not undergo any significant changes during life, although a great variability of the property is found in 13–14-aged children [7].

Many researchers highlight the absence of connection between enamel solubility and its resistance or susceptibility to caries, as well as between enamel solubility and the presence or absence of a carious lesion in the tooth.

According to V.A. Distal, (1975) [54], the solubility of the surface enamel layer in caries does not differ from that of the intact teeth. Even in the carious spot area, the caries enamel has the same solubility as the intact one. The author associates this phenomenon with the intense remineralization processes, which occurs in the surface layer of the enamel.

#### Composition of the saliva

One of the important functions of the saliva is to maintain a constant composition and properties of the enamel and to ensure its physical and chemical exchange with the external environment and the oral cavity. The study of saliva has shown that the mechanism of its mineralizing action is connected with its composition and properties. The main feature of saliva is its great oversaturation with Ca and P ions. The saliva is saturated with these ions 2.0–2.5 times more than the blood serum, which maintains enamel composition relatively constant [2].

Another property of the saliva is a significant preponderance of the P ions over the Ca ions in it. In blood serum, the Ca content is 9–11 mg/100 ml and the P content is 2–5 mg/100 ml; in saliva the Ca content is 3–6 mg/100 ml and the P content is 10–20 mg/100 ml. In physiological conditions (pH 7.0–7.5), it is about 50%, and most phosphate in saliva is ionized. Calcium in saliva appears mostly in a form of ions and phosphate—mainly in a form of hydrophosphate (HPO_4_^2−^) with a small part of dihydrophosphate (HPO^4−^) [6,55].

An important feature of the saliva composition is a simultaneous combination of soluble and stable calcium and phosphate ions. In conventional solutions, these ions are not compatible, they react and precipitate. According to the authors, the stability of the ions in saliva is explained by a number of reasons. The main one is the protective function of proteins, which in correlation to ionized calcium prevents from its reacting with phosphate and precipitating. Another reason for this fact is a significant preponderance of the phosphate content over calcium, which prevents from their equivalent interaction and removing from the reaction by precipitation [1].

#### Anatomical changes and features of ion-exchange tooth enamel

The structure of the enamel surface determines changes associated with age. The 7–14 age group is characterized by a marked macrorelief of the tooth surface, which often appears to be scalloped. The regular waviness of the enamel formed by perikymats is also typical. Teeth of the middle age group (20–40 years) are notable for a less marked relief of the enamel surface in comparison with those of children. By 20 years, perikymats become erased and may remain only in the cervical area. Later, they all disappear. On the occlusal surfaces of teeth, attrition sites appear. Their structure is non-prismatic as well as a part of the surface layer. The prisms contact is in the cervical and proximal areas with a typical elevation of their heads above the enamel surface [13].

In the over 45 age group, teeth are characterized by a significant attrition of dental cusps and incisal edges. The abrasion increases with age. On the surface of the intact enamel, a significant number of scratches, fissures, and cracks are revealed. Strips and scratches cross the tooth in different directions. The cracks are often located on the vestibular surface in parallel to the vertical axis of the tooth. They are normally filled with an amorphous substance [50].

A number of studies conducted at the Department of Therapeutic Dentistry of VSMA made it possible to determine that with age, there is a decline in the quality of metabolic processes of treated hard dental tissues. The microanalytical findings have revealed that patients of the “young” group have a rather high concentration of microelements (calcium and phosphorus) in the area bordering with a resin and even interpenetration of microelements from the filling material into hard dental tissues, and vice versa. In older age groups, such processes are less intensive [56].

Pathological and physiological abrasion occurs in dental hard tissues with age. These changes produce differences in mineral exchange in different age groups. Previous studies have reported changes in the structure of dental hard tissues in pathological abrasion. It is associated with the presence or absence of compensatory reactions of the tooth, which is confirmed by a number of surveys including radiograph descriptions. Where compensatory reactions are seen, an increased density of the enamel is observed, which indicates an increased ionic exchange activity of the enamel; if the compensatory reaction is absent, the enamel density is considerably lower. Acid biopsy findings determined different retention capacities of Ca and P. In the group with a compensatory reaction of the tooth, the Ca and P loss is less compared to the groups with no compensatory reaction [57].

This work defines two types of reactions, but there is no clear understanding about the age at which compensatory and non-compensatory reactions are found. Previous studies have revealed that age is an important factor influencing the structure and chemical composition of hard dental tissues.

Despite the fact that human enamel is the densest substance in the body, recent studies [42] have shown significant alterations its ionic exchange depending on a variety of variations. One of the varieties is the age of the person. Even today, there are still no statistically reliable results regarding the dependence of changes in organic and inorganic components of the enamel on age.

Various recent reports from the Department of Therapeutic Dentistry of VSMA have studied not only the structural-morphological features of enamel in children and adults but also the level of ionic exchanges inside it [9,42,56].

As a result of whole body aging, teeth change both the enamel structure and the level of its ionic exchanges; unfortunately, these changes have not been characterized in teeth with conventional age-specific differences.

#### Diagnostic biomarkers in saliva: noninvasive approach for prediction of aging processes

Saliva biomarkers are related to age features of teeth [58,59]. The saliva status during the different age periods is related to age features of tooth enamel. Therefore, a noninvasive approach to the prevention of age processes in the form of diagnostic saliva biomarkers has great therapeutic importance [60,61]. The use of salivary biomarkers has been useful in understanding of periodontal diseases [62,63]. Hopefully, salivary biomarkers will help in understanding enamel aging.

## Conclusions

There are insufficient data of morphological and microchemical changes in the enamel associated with age, both in normal and in carious enamel. It is indisputable that the comparison of these characteristics would contribute to the development and production of new hygienic and preventive anticarious products and filling materials for dental caries treatment in patients of different ages. Modern preventive dentistry makes a distinction between preventive and treatment agents meant for children and adults. But if the research results identify significant morphological and chemical changes in teeth of patients of different age, it will contribute to the planning of new perspective treatment materials that are optimized for different ages of patients.
